# Overall survival based on risk prediction scores after cardiac surgery in carcinoid heart disease

**DOI:** 10.1016/j.xjon.2025.03.024

**Published:** 2025-04-08

**Authors:** Mohamad S. Alabdaljabar, Tedy Sawma, S. Allen Luis, Juan A. Crestanello, Sorin V. Pislaru, Patricia A. Pellikka, Heidi M. Connolly

**Affiliations:** a Department of Internal Medicine, Mayo Clinic, Rochester, Minn; b Cardiovascular Surgery, Mayo Clinic, Rochester, Minn; c Department of Cardiovascular Medicine, Mayo Clinic, Rochester, Minn

**Figure:**
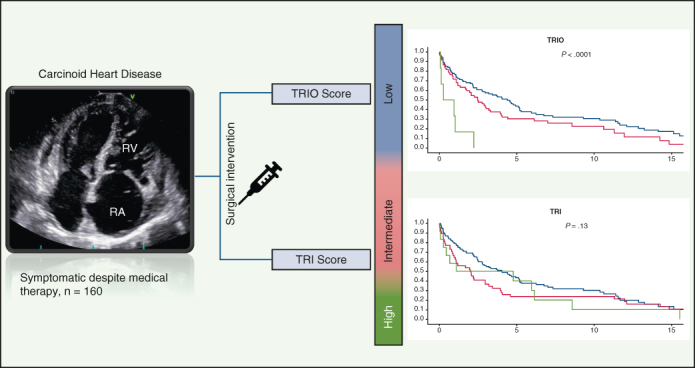
Postoperative survival of 160 patients who underwent tricuspid valve replacement for carcinoid heart disease. Survival stratified by outcome according to TRIO score and TRI-SCORE is shown.

**Central Message**

Carcinoid heart disease carries a high risk of morbidity and mortality, and surgical intervention also carries some risk. Our results suggest a lower postoperative survival rate in patients with a high TRIO score compared to those with low to intermediate scores.

Patients with neuroendocrine tumors commonly develop carcinoid heart disease (CaHD), which affects predominantly right-sided valves (mainly tricuspid).^1^ CaHD carries risks of mortality and morbidity, with a 3-year survival of ≤50% for untreated patients with severe cardiac symptoms. Surgical valve replacement carries some risk, and which patients benefit most from surgical intervention is unclear. Hence, in the present study, we aimed to evaluate survival after cardiac surgery based on available risk prediction scores and to identify patients who benefit most from surgical intervention.

Given that most CaHD patients have tricuspid regurgitation (TR),^2^ we retrospectively applied the Tricuspid Regurgitation Impact on Outcomes (TRIO)^3^ score and the TRI-SCORE^4^ to risk-stratify patients and to estimate the overall mortality. The TRIO score proposed to risk-stratify patients with TR is based on 8 parameters: age, sex, congestive heart failure (CHF), chronic lung disease, heart rate, creatinine, aspartate transaminase, and presence of severe TR. The TRI-SCORE was developed to predict in-hospital mortality after isolated tricuspid valve surgery and comprises 13 variables: age, sex, New York Heart Association class, right-sided CHF signs, prior left valvular intervention, pacemaker/defibrillator, atrial fibrillation/flutter, furosemide dose, glomerular filtration rate, total bilirubin, left ventricular ejection fraction (LVEF), right ventricular (RV) dysfunction, and TR mechanism. The TRIO score was developed across all TR groups, including CaHD patients.

Survival from the time of surgical intervention was evaluated using Kaplan-Meier (KM) and Cox regression methods based on the Social Security Death Index. Statistical analyses were performed using BlueSky v10.2.0 (BlueSky Statistics).

Using the Mayo Clinic cardiac surgical database, we included patients with (1) an International Classification of Diseases Ninth/10th Revision diagnosis of neuroendocrine tumor, (2) echocardiographic evidence of CaHD, and (3) surgical intervention for CaHD at Mayo Clinic between 2001 and 2019. The study was approved by the Institutional Review Board (ID 21-013364; approved January 18, 2022), and all included patients provided informed consent. Comprehensive echocardiography was performed based on guidelines.^5^

The study cohort of 160 patients included 87 females (52%) and had a median age of 63.8 years (range, 56.0-69.6 years). The prevalence of CHF was 36.3% (58 patients); 107 patients (66.9%) were on a diuretic, with a median dose of furosemide (or equivalent) of 20 mg (range, 0-40 mg). On echocardiography, moderate or greater right atrial or RV enlargement was present in 72.5% and 76.9%, respectively, and RV dysfunction was present in 13.1%. The most common valvular disease of at least moderate severity was TR (100%), followed by pulmonary regurgitation (82.5%). One-fifth of the cohort had an RV systolic pressure >50 mm Hg, and 6.9% had an LVEF <50%. Thirty-three patients (20.6%) had left-sided disease (valvular disease of moderate or greater severity or LVEF <50%). Most patients had low TRIO/TRI scores (58.8/62.5%), 37.5/30.0% had intermediate scores, and 3.8/7.5% had high scores.

All patients underwent tricuspid valve replacement, and 79.4% also had pulmonic valve replacement. Other interventions included aortic and mitral valve repair/replacement (11% and 13%, respectively), patent foramen ovale closure/atrial septal defect repair (31%), and coronary artery bypass (7%). The median survival after intervention was 3.0 years (range, 2.3 to 4.4 years).

On Kaplan-Meier graphs (Figure 1), both TRIO score and TRI-SCORE were associated with overall mortality risk. The median survival by TRIO score (*P* < .0001) was 4.4 years (95% CI, 2.9-5.3 years) for low, 2.5 years (95% CI, 1.7-4.0 years) for intermediate, and 0.6 year (95% CI, 0.1 year to not available) for high scores, and respective TRI-SCORE values (*P* = .13) were 4.2 years (95% CI, 2.8-5.2 years), 1.9 years (95% CI, 1.1-3.4 years), and 2.9 (95% CI, 0.4 year to not available). On Cox regression, the hazard ratio (HR; per risk group change) was 1.64 (95% CI, 1.19-2.27; *P* = .002) for TRIO score and 1.29 (95% CI, 0.99-1.67; *P* = .054) for TRI-SCORE. This was true and remained unchanged even after excluding patients with left-sided disease, after which 4 patients remained in the high TRIO group and 10 patients remained in the high TRI-SCORE group. Among the 6 patients with a high TRIO score, 4 died from metastatic carcinoid disease or carcinoid crisis and 2 died from cardiac causes. Among 12 patients with high TRI-SCORE, 4 patients likely died from carcinoid disease; 1 died due to postoperative bleeding, ventricular tachycardia, and multiorgan failure; and the remainder died due to unclear reasons.

**Figure 1:**
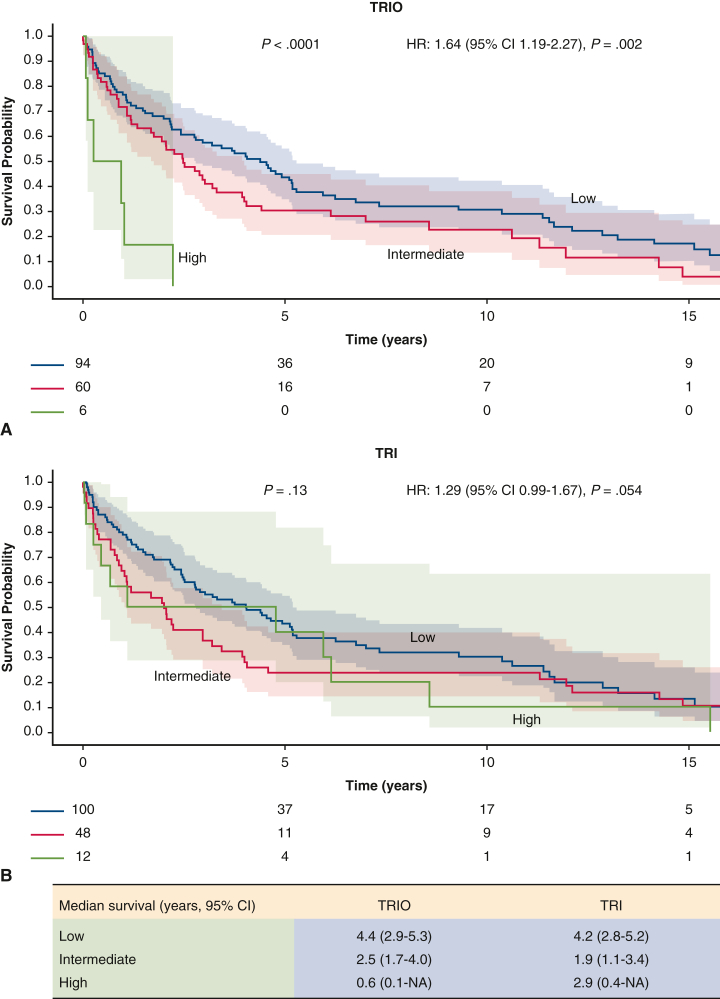
Survival in 160 patients with carcinoid heart disease who underwent surgical intervention from the time of intervention to last follow up based on the Tricuspid Regurgitation Impact on Outcomes (*TRIO*) score (A) and the TRI-SCORE (B) with 95% confidence intervals. TRIO performed better in differentiating patients according to postoperative outcome.

As opposed to low/intermediate TRIO score, patients with a high TRIO score did not seem to have a survival advantage with surgical intervention, with a median survival rate of <1 year. Both the TRIO score and TRI-SCORE correlated with mortality, and the TRIO score demonstrated better separation for patients with worse risk scores. The median survival of the cohort was 3.0 years, which is comparable to previous reports.^1^^,^^2^

The TRIO score provided more reliable risk stratification than the TRI-SCORE, particularly in patients with intermediate to high scores. Based on this analysis, CaHD patients with high TRIO score might not benefit as much as other patients from surgical intervention; however, this result should be interpreted cautiously, given the low number of patients in the high TRIO score group. Progression of underlying metastatic disease, severe carcinoid syndrome, cardiac dysfunction, and comorbid disorders likely explain the lack of survival benefit in the high-risk TRIO score group. In the absence of guidelines for CaHD management, our study shows that TRIO score may be helpful for surgical risk stratification and patient selection.

Limitations of this study include its single-center study and retrospective and observational nature. Although both risk scores used were intended for TR risk stratification, patients with CaHD being considered for surgical intervention have severe TR and often pulmonary regurgitation and/or other valve disorders. CaHD patients should be evaluated for surgical intervention prior to disease progression (ie, high TRIO score) for better results. Overall mortality and quality of life are dependent not only on cardiac function, but also on the stage/progression of the tumor and treatment. These factors should be included when discussing management options and overall prognosis for CaHD patients.

## Conflict of Interest Statement

The authors reported no conflicts of interest.

The *Journal* policy requires editors and reviewers to disclose conflicts of interest and to decline handling or reviewing manuscripts for which they may have a conflict of interest. The editors and reviewers of this article have no conflicts of interest.
